# Desminopathy: Novel Desmin Variants, a New Cardiac Phenotype, and Further Evidence for Secondary Mitochondrial Dysfunction

**DOI:** 10.3390/jcm9040937

**Published:** 2020-03-29

**Authors:** Miloš Kubánek, Tereza Schimerová, Lenka Piherová, Andreas Brodehl, Alice Krebsová, Sandra Ratnavadivel, Caroline Stanasiuk, Hana Hansíková, Jiří Zeman, Tomáš Paleček, Josef Houštěk, Zdeněk Drahota, Hana Nůsková, Jana Mikešová, Josef Zámečník, Milan Macek, Petr Ridzoň, Jana Malusková, Viktor Stránecký, Vojtěch Melenovský, Hendrik Milting, Stanislav Kmoch

**Affiliations:** 1 Department of Cardiology, Institute for Clinical and Experimental Medicine, 14021 Prague, Czech Republic; nedt@ikem.cz (T.S.); krea@ikem.cz (A.K.); vome@ikem.cz (V.M.); 2 Institute of Physiology, First Faculty of Medicine, Charles University, 11636 Prague, Czech Republic; 3 Research Unit for Rare Diseases, Department of Pediatrics and Adolescent Medicine, First Faculty of Medicine, Charles University, 11636 Prague, Czech Republic; Lenka.Piherova@lf1.cuni.cz (L.P.); Viktor.Stranecky@lf1.cuni.cz (V.S.); skmoch@lf1.cuni.cz (S.K.); 4 Erich and Hanna Klessmann Institute, Heart and Diabetes Center NRW, University Hospital of the Ruhr-University Bochum, 32545 Bad Oeynhausen, Germany; ABrodehl@hdz-nrw.de (A.B.); SRatnavadivel@hdz-nrw.de (S.R.); CStanasiuk@hdz-nrw.de (C.S.); HMilting@hdz-nrw.de (H.M.); 5 Department of Pediatrics and Adolescent Medicine, First Faculty of Medicine, Charles University and General University Hospital in Prague, 12108 Prague, Czech Republic; Hana.Hansikova@lf1.cuni.cz (H.H.); jzem@lf1.cuni.cz (J.Z.); 6 2nd Department of Medicine–Department of Cardiovascular Medicine, First Faculty of Medicine, Charles University and General University Hospital in Prague, 12108 Prague, Czech Republic; Tomas.Palecek@lf1.cuni.cz; 7 Institute of Physiology, Czech Academy of Sciences, 11720 Prague, Czech Republic; josef.houstek@fgu.cas.cz (J.H.); zdenek.drahota@fgu.cas.cz (Z.D.); h.nuskova@dkfz-heidelberg.de (H.N.); jana.mikesova@uochb.cas.cz (J.M.); 8 Department of Pathology and Molecular Medicine, Second Faculty of Medicine, Charles University, 11636 Prague, Czech Republic; josef.zamecnik@lfmotol.cuni.cz; 9 Department of Biology and Medical Genetics, Second Faculty of Medicine, Charles University, 11636 Prague, Czech Republic; milan.macek.jr@lfmotol.cuni.cz; 10 Department of Neurology, Thomayer’s Hospital, 14059 Prague, Czech Republic; petr.ridzon@ftn.cz; 11 Department of Pathology, Institute for Clinical and Experimental Medicine, 14021 Prague, Czech Republic; Institute for Clinical and Experimental Medicine, 14021 Prague, Czech Republic; jana.maluskova@ikem.cz

**Keywords:** desmin, dilated cardiomyopathy, mitochondrial dysfunction, myopathy, non-ischemic cardiomyopathy, whole exome sequencing

## Abstract

**Background:** The pleomorphic clinical presentation makes the diagnosis of desminopathy difficult. We aimed to describe the prevalence, phenotypic expression, and mitochondrial function of individuals with putative disease-causing desmin (DES) variants identified in patients with an unexplained etiology of cardiomyopathy. **Methods:** A total of 327 Czech patients underwent whole exome sequencing and detailed phenotyping in probands harboring DES variants. **Results:** Rare, conserved, and possibly pathogenic DES variants were identified in six (1.8%) probands. Two DES variants previously classified as variants of uncertain significance (p.(K43E), p.(S57L)), one novel DES variant (p.(A210D)), and two known pathogenic DES variants (p.(R406W), p.(R454W)) were associated with characteristic desmin-immunoreactive aggregates in myocardial and/or skeletal biopsy samples. The individual with the novel DES variant p.(Q364H) had a decreased myocardial expression of desmin with absent desmin aggregates in myocardial/skeletal muscle biopsy and presented with familial left ventricular non-compaction cardiomyopathy (LVNC), a relatively novel phenotype associated with desminopathy. An assessment of the mitochondrial function in four probands heterozygous for a disease-causing DES variant confirmed a decreased metabolic capacity of mitochondrial respiratory chain complexes in myocardial/skeletal muscle specimens, which was in case of myocardial succinate respiration more profound than in other cardiomyopathies. **Conclusions:** The presence of desminopathy should also be considered in individuals with LVNC, and in the differential diagnosis of mitochondrial diseases.

## 1. Introduction

Desminopathy (OMIM # 601409) represents a group of autosomal inherited disorders caused by pathogenic variants in the disease-causing desmin (*DES*) gene, encoding the major muscle specific intermediate filament protein desmin (OMIM: #125660) [1]. Desmin is the major component of intermediate filaments in cardiac, skeletal, and smooth muscle cells, with a particularly high content in Purkinje fibers and diaphragmatic muscle cells [1]. Consequently, cardiomyopathy, cardiac conduction disease, and progressive skeletal myopathy are the most common clinical presentations of desminopathy. It may occur as an isolated cardiac disease or in variable combinations and with different onsets. As summarized in a meta-analysis [1], 49%, 60%, and 74% of individuals harboring a pathogenic *DES* variant develop cardiomyopathy, cardiac conduction disease, and skeletal myopathy, respectively. The most common form of myocardial involvement is dilated cardiomyopathy (DCM) [1,2,3], followed by restrictive (RCM) [4,5,6,7], arrhythmogenic (ACM) and hypertrophic cardiomyopathy (HCM), and arrhythmogenic cardiomyopathy pattern [8,9,10,11]. On the other hand, there is low evidence regarding an association between desminopathy and left ventricular noncompaction cardiomyopathy (LVNC). Importantly, intermediate filaments are essential not only for cellular integrity, organization, and differentiation, but also for a signal transduction and adequate mitochondrial function [12]. Accordingly, several experimental [12,13,14] and clinical [15,16] studies have proven a secondary mitochondrial dysfunction in desminopathy, which in one case even mimicked mitochondrial disease [16].

The pleomorphic clinical presentation makes the diagnosis of desminopathy challenging. Fortunately, massively parallel sequencing (MPS) utilizing either cardiomyopathy panels and/or even whole exome sequencing (WES) aid in the diagnosis of desminopathy regardless of its clinical presentation. Hereby, we aimed to describe the prevalence of desminopathy and their phenotypes in a large representative cohort of patients with cardiomyopathy of unexplained etiology using WES.

## 2. Materials and Methods

**Figure 1:**
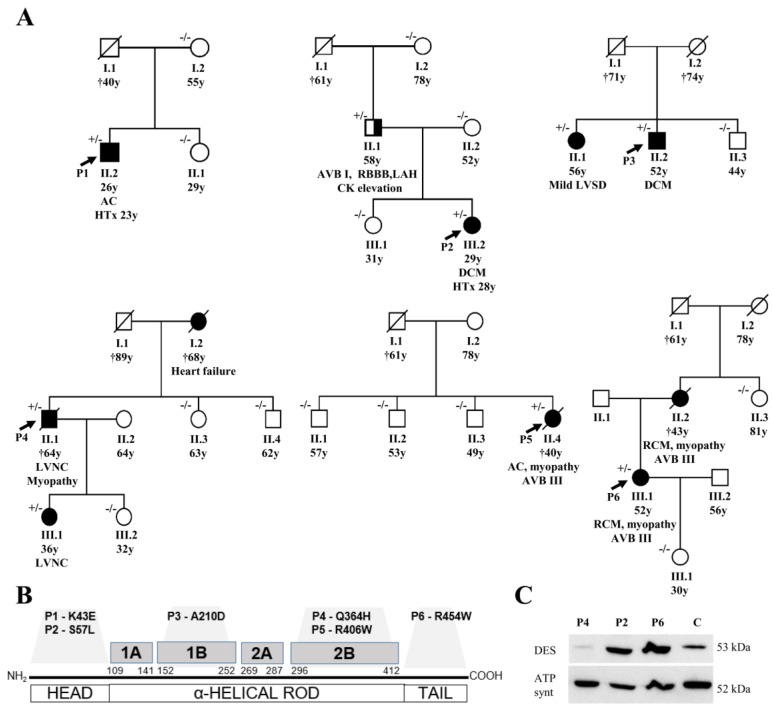
(**A**) shows pedigrees of the affected families and segregation of desmin variants (+/− heterozygous status, −/− wild type). In the fourth family (with P4) we also assessed a segregation of the rare variant of *MYH7* (NM_000257.3), c.4679G > C, p.(Arg1560Pro), which was present just in P4 (II/1) and absent in II/3, II/4, III/1, and III/2. (**B**) summarizes the structure of the desmin gene with localization of the detected variants. (**C**) illustrates the detection of desmin by western blot in myocardial samples (P2, P4, P6, control sample; 30ug protein aliquots) with an obvious reduction of signal in P4 with left ventricular non-compaction cardiomyopathy (*DES*-p.(Q364H)). Abbreviations: AC = arrhythmogenic cardiomyopathy, AVB = atrioventricular block, DCM = dilated cardiomyopathy, DES = desmin, HTx = heart transplantation, LAH = left anterior hemiblock, LVNC = left ventricular non-compaction cardiomyopathy, LVSD = left ventricular systolic dysfunction, RBBB = right bundle branch block, and RCM = restrictive cardiomyopathy.

A representative cohort of 327 Czech patients with an unexplained etiology of cardiomyopathy underwent WES between September 2015 and June 2017. The cohort consisted mainly of cases with familial and sporadic DCM (81%), LVNC (13%), and less frequently of RCM (6%) or ACM (6%). Rare and possibly pathogenic missense *DES* variants were identified in 6 (1.8%) index patients from 6 different families (Figure 1).

### 2.1. Clinical Description of Studied Patients and of Their Families

Comprehensive clinical, laboratory, and electrophysiological data of all index cases were collected. Two probands of them (P2, P4) also underwent cardiovascular magnetic resonance imaging (Siemens Trio scanner, Siemens Medical Solutions, Erlangen, Germany) as described previously [17]. All available relatives undertook cardiologic screening, which included physical examination, electrocardiography, and echocardiography as well as a collection of blood samples for genetic analysis. Patients with suspected disease-causing *DES* variants were subjected to a detailed neurologic assessment, measurement of serum creatine phosphokinase, nerve conductance, and electromyography of two muscles (left vastus medialis and left deltoid muscle), as reported previously [17].

The study was approved by the Institutional Review Board´s representing all clinical collaborators (Institute for Clinical and Experimental Medicine and Thomayer´s Hospital; 1st Faculty of Medicine of the Charles University and General Faculty Hospital; both Prague) and was conducted in accordance with the principles of the Declaration of Helsinki. Written informed consent was obtained from all probands.

### 2.2. Genetic Analysis and Detection of Variants

To detect causal genetic variants, WES was performed according to internationally accepted guidelines [18]. Full technical details are provided in the Supplementary Materials. The criteria for classifying variants as putative disease-causing variants included their rare occurrence (≤0.05% among control samples), changes in predicted amino acid sequences, conservation across different species (http://www.ncbi.nlm.nih.gov/BLAST/), segregation within the family, and previously reported pathogenicity in databases.

Exons with identified variants of the *DES* gene were PCR amplified (Table S1) from genomic DNA of all available individuals from the analyzed families and sequenced using the version 3.1 Dye Terminator cycle sequencing kit with electrophoresis on an ABI 3500XL Avant Genetic Analyzer (both ThermoFisher Scientific; Waltham, MA, USA). Data were analyzed using Sequencing Analysis software version 6.0 (both ThermoFisher Scientific; USA) and the segregation of the candidate *DES* variants with the phenotype was evaluated.

### 2.3. In Vitro Analysis of DES Variants

As many but not all pathogenic *DES* variants cause an abnormal cytoplasmic desmin aggregation, we constructed for the identified *DES* variants expression plasmids by site-directed mutagenesis (Agilent Technologies, Santa Clara, CA, USA) according to the manufacturer’s instructions. Desmin encoding parts of all plasmids were verified by Sanger sequencing (Macrogen, Amsterdam, Netherlands). The plasmid pmRuby-N1-DES and pmRuby-N1-DES-p.(Y122C) have been previously described [19,20]. Previously reported variant DES-p.(Y122C) was used as a positive control forming abnormal cytoplasmic aggregates [20]. HT1080 cells, which do not express endogenous desmin and cardiomyocytes derived from human induced pluripotent stem cells (iPSC) (NP00040-8) were transfected using Lipofectamin 3000 (ThermoFisher Scientific) or nucleofection using the 4D Nucleofector (Lonza, Cologne, Germany) in combination with the P3 Primary Cell 4D Nucleofector Kit according to the manufacturer’s instructions. The differentiation of hiPSCs has been previously described [21]. Transfected HT1080 cells were fixed using 4% paraformaldehyde, permeabilized using 0.05% Triton X100, and stained with phalloidin conjugated with Alexa-488. Transfected hiPSC-derived cardiomyocytes were stained with primary antibodies against the Z-band protein α-actinin as a cardiomyocytes specific marker (Sigma-Aldrich, Missouri, MO, USA, #A7732) in combination with secondary antibodies conjugated to Alexa-488 (ThermoFisher). Confocal microscopy was performed as previously described [22].

### 2.4. Statistical Analysis of Aggregate Formation

A total of 3 to 4 independent transfection experiments were analyzed by counting the number of aggregate forming cells. Non-parametric Kruskal–Wallis for multiple comparison was performed using GraphPad Prism version 8.3.0 for Windows (GraphPad Software, San Diego, CA, USA). *p*-values <0.05 were considered as significant.

### 2.5. Histopathology, Immunohistochemistry, Desmin Western Blot, and Electron Microscopy

In 5 probands (P1–P4, P6), formalin-fixed paraffin-embedded samples of myocardium were available either from endomyocardial biopsy (P2, P3) and/or from hearts explanted during transplantation (P1, P2, P6) or post-mortem (P4). The samples were snap frozen in liquid nitrogen and stored at −70 °C. Resin-embedded myocardial samples for electron microscopy were analyzed in 4 patients (P1, P2, P4, and P6). A biopsy of skeletal muscle was performed in 3 individuals with clinical signs of myopathy (P4 and P5: Soleus-, P6: Deltoid muscle). In P4, also we obtained samples of intercostal muscles post-mortem. The excisions from the skeletal muscle (approx. 10 × 5 × 5 mm in size) were snap frozen in isopentane (2-methylbutane; Merck, Kenilworth, NJ, USA) and cooled in liquid nitrogen. Cryosections were examined by routine hematoxylin–eosin staining and a conventional spectrum of histochemical reactions, including myofibrillary ATPase, nicotinamide adenine dinucleotide-tetrazolium reductase (NADH-TR), succinate dehydrogenase (SDH), and cytochrome c oxidase (COX), as described elsewhere [23].

Desmin immunohistochemistry and electron microscopy were performed on both skeletal muscle and myocardium samples according to standard protocols (Supplementary Materials).

### 2.6. Analysis of Mitochondrial Function in Biopsies

Skeletal muscle homogenate (5%, w/v) was prepared from fresh tissue by using a glass-Teflon homogenizer in a medium containing 150 mM KCl, 50 mM Tris-HCl, 2 mM EDTA, pH 7.4, and 0.2 ug/mL Aprotinin at 4 °C. Mitochondria were isolated from the homogenate by differential centrifugation as described elsewhere [24]. Heart tissue homogenates (7%, w/v) were prepared from −80 °C stored frozen samples of left and right heart ventricles in 0.32 M sucrose, 10 mM Tris-HCl, 1 mM EDTA, pH 7.4, and 1μg/mL PIC (protease inhibitor mixture Sigma P8340) using glass-Teflon and glass-glass Dounce homogenizers. The subsequent methods are described in detail in Supplementary Materials. A western blot analysis of mitochondrial proteins, measurement of mitochondrial DNA content, measurement of activities of respiratory chain complexes and citrate synthase [25], high resolution oxygraphy, and measurement of the content of total coenzyme Q10 were described previously in details and in the Supplementary Materials.

## 3. Results

### 3.1. Description of DES Variants and Their Segregation in Families

**Figure 2:**
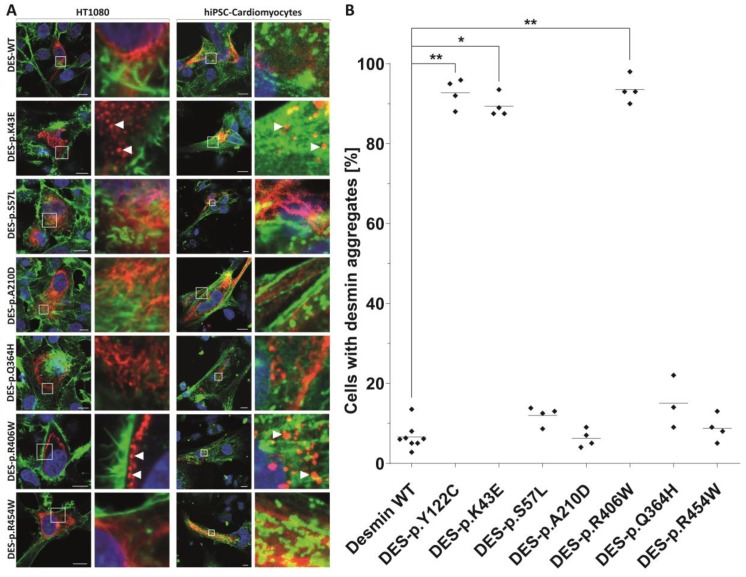
Cell transfection experiments of transfected HT1080 cells and iPSC-derived cardiomyocytes. Mutant and wild-type desmin was expressed with the red fluorescent protein-tag mRuby at the C-terminus (shown in red). Representative confocal images are shown (**A**). In case of HT1080 cells, F-actin was stained using phalloidin-Alexa488 (shown in green) and the nuclei were stained using 4′,6-diamidin-2-phenylindole (shown in blue). In case of iPSC-cardiomyocytes, the cardiomyocyte marker α-actinin was stained using antibodies (shown in green) and the nuclei were stained with DAPI (shown in blue). Scale bars represent 10 µm. (**B**) Quantification of aggregate formation was performed in three to four independent transfection experiments of HT1080 cells. * *p* < 0.05 and ** *p* < 0.01. The variant DES-p.(Y122C) was used as a positive control forming abnormal cytoplasmic aggregates [20].

Probably disease-causing *DES* variants in heterozygous constitution were identified in six index cases (1.8%). Two missense variants were identified within the non-helical head (amino-teminal) domain of desmin, i.e., in P1 with biventricular form of ACM (NM_001927.3: c.127A > G; NP_001918.3: p.(K43E)) and in P2 with DCM (NM_001927.3: c.170C > T; NP_001918.3: p.(S57L)) (Figure 1, Tables S2 and S3). Both of them were previously reported in Clinvar database as variants of uncertain significance. In addition, we analyzed the desmin filament formation in transfected HT1080 and in iPSC-derived cardiomyocytes, revealing an abnormal cytoplasmic aggregation in the DES-p.(K43E) variant and known pathogenic DES-p.(R406W) variant (Figure 2). Two novel variants were found in the highly conserved central α-helical rod domain, i.e., in P3 with familial DCM located in the 1B helical domain (NM_001927.3:c.629C > A; NP_001918.3: p.(A210D)) and in P4 with familial LVNC in combination with skeletal myopathy located in the 2B helical domain (NM_001927.3: c.1092G>T; NP_001918.3: p.(Q364H)) (Figure 1, Tables S2 and S3). The findings in the biopsies are described below. The remaining two probands had the following known *DES* pathogenic variants: P5 with ACM and skeletal myopathy in the 2B helical domain (NM_001927.3: c.1216C>T; NP_001918.3: p.(R406W); HGMD database ((http://www.hgmd.cf.ac.uk/ac/index.php) CM000368) [6] and P6 with RCM and skeletal myopathy within the non-helical tail (carboxy-terminal) domain (NM_001927.3: c.1360C > T; NP_001918.3: p.(R454W); HGMD CM071700) [26] (Figure 1, Tables S2 and S3). Table S4 contains lists of rare genetic variants of further cardiomyopathy associated genes in all probands (frequency in Exac database less than 0.00001). Just the variant of *MYH7* (NM_000257.3) c.4679G > C, p.(Arg1560Pro) in proband 4 could be relevant in a patient with LVNC. However, it was not present in other members of the family tested (II 1, 3, 4; III 1, 2) (Figure 1) and did not co-segregate with the phenotype of LVNC. Importantly, any pathogenic variants in mitochondrial proteins coded by nuclear DNA or mitochondrial DNA were not found in these six probands.

Figure 1 illustrates the segregation of *DES* variants in families. Family history or clinical screening revealed a similar cardiac disease in a first-degree relative in P3, P4, and P6 segregating with occurrence of *DES* variants (Figure 1, Table S2). In the father of P2, heterozygous for *DES* p.(S57L) variant, we observed an incomplete penetrance of the disease with atrioventricular block grade I, right bundle branch block, left anterior hemiblock, normal echocardiography, and a mild elevation of creatinine phosphokinase of 6.1 μkat/l (upper limit of normal 2.3 μkat/L) without clinical signs of myopathy. Cases P1 and P5 seemed to be sporadic (segregation assessed in mother and sister of P1, and three siblings of P5).

### 3.2. Phenotypes of Desminopathy

**Figure 3:**
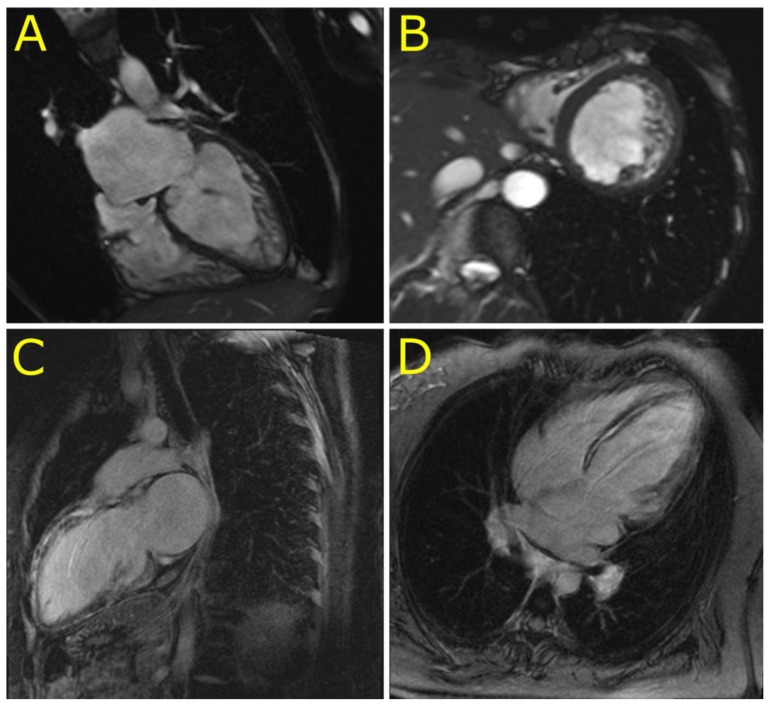
Cardiovascular magnetic resonance imaging in patients with left ventricular non-compaction cardiomyopathy (P4) and dilated cardiomyopathy with an extensive late gadolinium enhancement (P2). (**A**,**B**): Four chamber and short axis views of left ventricular non-compaction cardiomyopathy in P4. (**C**,**D**): Two chamber long axis and four chamber views of an extensive late gadolinium enhancement in the ventricular septum and left ventricular anterior wall mimicking inflammatory cardiomyopathy in P2.

The initial clinical presentation included cardiac arrest due to ventricular tachycardia in the 2nd decennium (P1), complete atrioventricular blockade in the 3rd decennium (P5, P6), and heart failure in the 3rd to 5th decennium (P2, P3, and P4). Skeletal myopathy and dysfunction of bulbar muscles became apparent during the 4th to 6th decennium in cases 4–6 (Tables S2 and S3). An unusual clinical presentation had proband 2. A young female presented with acute heart failure, a severe systolic dysfunction of mildly dilated left ventricle, persistent elevation of troponin T (> 10 times the upper limit of normal) (Table S3), and an extensive mid-wall late gadolinium enhancement of the septum and anterior wall of the left ventricle (Figure 3). These findings mimicked inflammatory cardiomyopathy however, there was no sign of inflammation as assessed by endomyocardial biopsy. Inflammation was absent also in her heart explanted during transplantation three years later. The arrhythmogenic left ventricular cardiomyopathy was considered as an alternative diagnosis in P2. However, her electrocardiogram was unremarkable and ventricular extrasystoles were infrequent. Proband 4 presented with a unique phenotype of LVNC. Magnetic resonance imaging (Figure 3) confirmed the diagnosis of LVNC with a percentage of non-compaction within the total left ventricular mass of 43%. Proband 6 was incorrectly diagnosed with mitochondrial disease based on skeletal muscle biopsy performed several years ago. This diagnosis was reclassified to desminopathy after the identification of known pathogenic desmin mutation (p.(R454W)) and morphological analysis of myocardial samples from the explanted heart. Table S3 illustrates additional clinical and laboratory data of the study group including echocardiography. During a median follow-up of 56 months (31–182), five probands (83%) developed end-stage heart failure.

### 3.3. Morphology of Desminopathy in Myocardial and Skeletal Muscle Samples

**Figure 4:**
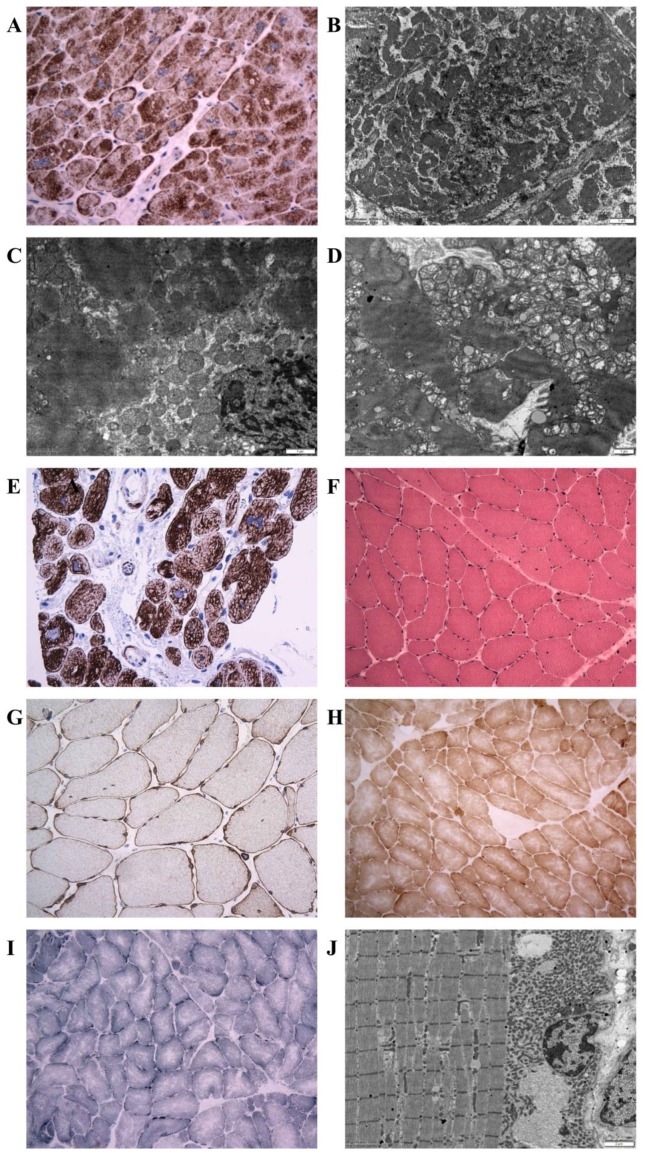
Illustration of histopathology, immunohistochemistry, and electron microscopy in individuals with the novel desmin variants. (**A**): Desmin immunohistochemistry (left ventricular myocardium, explanted heart, P1) documenting a diffuse alteration of desmin distribution with a formation of desmin aggregates revealing strong immunoreactivity in the cytoplasm. Original magnification ×400. (**B**): Electron microscopy (left ventricular myocardium, explanted heart, P1) detects amorphous granulofilamentous material in the cytoplasm of cardiomyocytes compatible with desmin aggregates. Original magnification ×10,000. (**C**,**D**): Electron microscopy (left ventricular myocardium, explanted heart, P2). (**C**): Pathological dense granulofilamentous inclusions in the cytoplasm of cardiomyocyte. Original magnification ×12,000. (**D**): Increased number of mitochondria in cardiomyocyte, often in clusters, with altered distribution. Original magnification ×8000. (**E**): Desmin immunohistochemistry (right ventricular myocardium, endomyocardial biopsy, P3) revealed an abnormal staining of cardiomyocytes with a formation of desmin positive aggregates. Original magnification ×400. (**F**–**J**) Diagnostic skeletal muscle biopsy specimens, Soleus muscle, P4, original magnification ×400. (**F**): By light microscopy with hematoxyllin-eosin, there was a marked variability in fiber size, absent inclusions, and increased number of internal nuclei. (**G**): Desmin immunohistochemistry did not reveal any protein aggregates in the sarcoplasma. (**H**): Nicotinamide adenine dinucleotide (NADH) and succinate dehydrogenase (SDH) immunohistochemistry identified few muscle fibers with increased oxidative activity at their periphery, indicating the pathological accumulation of mitochondria. However, no typical ragged red fibers were observed. (**I**): Very few COX-negative fibers were also present. (**J**): Electron-microscopic analysis revealed increased number of mitochondria, often in clusters, with altered distribution. No accumulation of intermediate filaments was observed. Original magnification ×6000.

An immunohistochemical examination of myocardial samples in P1–P3 and P6 showed a diffuse alteration of desmin distribution in cardiomyocytes with a formation of desmin aggregates revealing strong immunoreactivity in the cytoplasm (shown in P1, P3; Figure 4A,E). Electron microscopy of cardiomyocytes in P1, P2, and P6 revealed myofibrillar disruption, streaming Z bands, and deposits of dense, amorphous granulofilamentous material of variable size and shape (shown in P1, P2; Figure 4B,C). In addition, we constructed a set of expression plasmids for the six *DES* missense variants and transfected HT1080 as a cell model without endogenous desmin expression and iPSC-derived cardiomyocytes. These experiments revealed a severe intermediate filament formation defect for *DES*-p.(K43E) and *DES*-p.(R406W) underlining their pathogenicity. Furthermore, electron microscopy of cardiac tissue demonstrated in P2, P4, and P6 focally increased the number of mitochondria, often in clusters, with loss of mitochondrial spatial organization (P2; Figure 4D). Importantly, desmin aggregates were absent in myocardial samples of P4 both at immunohistochemical and ultrastructural analysis. An expression of desmin in myocardium (P2, P4, and P6) was also assessed by Western blot analysis. There was an obvious reduction of the signal in P4 (Figure 1B).

Samples of the skeletal muscle (P4–P5 m. soleus, P4 intercostal muscle, P6 m. deltoideus) showed different findings in P4 and P6 as compared with P5. The morphological analysis in P4 and P6 detected only mild myopathic changes. The light microscopy with hematoxylin-eosin staining showed a marked variability in fiber size and increased number of internal nuclei (P4; Figure 4F). No inclusions were observed by light microscopy. Similarly, desmin immunohistochemistry did not reveal any protein aggregates in the sarcoplasma of P4 and P6 (P4; Figure 4G). In the NADH and SDH reactions, many fibers did not possess the characteristic checkerboard pattern, and in a proportion of fibers there was increased oxidative activity at the periphery of the muscle fibers, indicating the pathological accumulation of mitochondria (P4; Figure 4H). However, no typical ragged red fibers were observed. The distribution of COX reactivity was altered similarly to a NADH/SDH pattern with very few COX-negative fibers present (P4; Figure 4I). On the other hand, the muscle biopsy in P5 showed severe myopathic changes with a large amount of fibro-fatty tissue in the interstitium of the muscle. Desmin immunohistochemistry confirmed in P5 a diffuse alteration of desmin distribution with a formation of desmin aggregates in the cytoplasm of muscle fibers.

An ultrastructural analysis of skeletal muscle biopsies revealed a focally increased number of mitochondria, often in clusters, with an altered distribution in P4 and P6 (P4; Figure 4J) however, no ultrastructural abnormality in mitochondria morphology was observed. Typical deposits of dense granulofilamentous material were absent in P4 and were not observed in P6 at the first reading. Thus the first description of the skeletal muscle biopsy in P6 led to the diagnosis of mitochondrial myopathy. Nevertheless, the second reading of the skeletal muscle biopsy performed with the knowledge of the results of genetic tests and abnormal immunostaining of desmin in myocardium discovered a focus of dense amorphous material in a single fiber at electron microscopy (not shown).

### 3.4. Indications for the Pathogenicity of the Novel Desmin Variants

A typical myocardial histopathology and ultrastructure with pathological desmin-immunoreactive aggregates strongly supported the pathogenicity of desmin variants p.(K43E), p.(S57L), and p.(A210D). In addition, the desmin filament formation experiments in transfected HT1080 and in iPSC-derived cardiomyocytes revealed an abnormal cytoplasmic aggregation of DES-p.(K43E). On the other hand, the pathogenicity of the novel desmin variant p.(Q364H) is supported mainly by decreased myocardial desmin expression and co-segregation of the above desmin variant in the family in the absence of other segregating cardiomyopathy-related genes as assessed by WES in the proband.

### 3.5. Mitochondrial Function and Content in Skeletal Muscle and Heart

**Figure 5:**
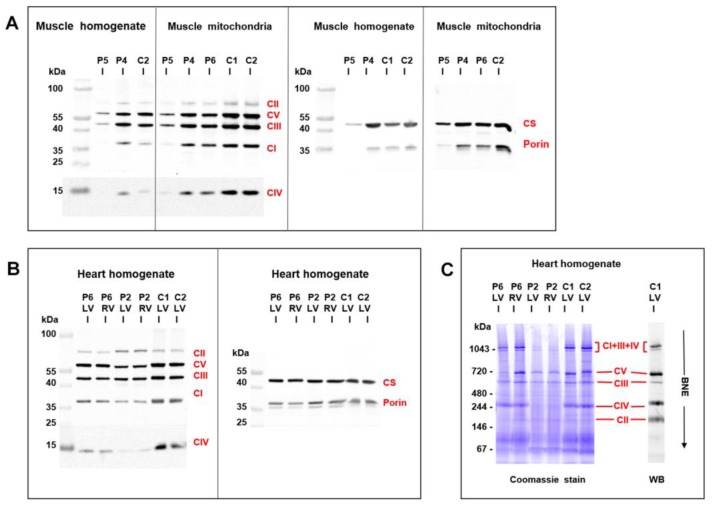
Western blot detection of mitochondrial proteins in skeletal muscle and heart. Analysis of SDS-PAGE resolved proteins from (**A**) muscle homogenates (6 µg protein aliquots) and isolated muscle mitochondria (2 µg protein aliquots) demonstrated a pronounced decrease of respiratory chain complexes (CI-CV), citrate synthase (CS), and porin in skeletal muscle of P5 compared to controls (**C**), P4 and P6 were less affected. Analysis of (**B**) heart homogenates (4 µg protein aliquots) from left and right heart ventricles (LV, RV) demonstrated a marked decrease of respiratory chain enzymes in P2 and a mild decrease in P6 compared to controls (**C**). CS and porin were less affected. BlueNative electrophoresis (**C**) further showed a marked decrease of native respiratory supercomplexes consisting of CI + CIII + CIV in P2 heart ventricles (12 µg protein aliquots).

An analysis of mitochondrial respiratory enzymes in skeletal muscle homogenates (Table 1) revealed a decreased activity of citrate synthase in P5, the activity of respiratory chain complex IV, and the quantity of mitochondrial respiratory chain proteins were decreased (Figure 5A). An oxygraphy analysis of P6 skeletal muscle fibers further showed a decrease in coupled (state 3-ADP) oxidation of NADH-dependent substrates (pyruvate + malate) to 35% of the mean of the controls (Table 1). More consistent data were provided by the analysis of isolated mitochondria from skeletal muscles of P4–P6. Specific activities of respiratory chain complexes I + III (NADH: Cytochrome c reductase), complex IV (cytochrome c oxidase), and citrate synthase were decreased to 30%–50% of the mean of the controls. Both P5 and P6 had a decreased content of coenzyme Q (Table 1). A low specific content of respiratory chain enzymes, citrate synthase, and porin was further apparent in isolated muscle mitochondria of P4–P6, with the most pronounced decrease observed in P5 (Figure 5A).

Myocardium of two patients with desminopathy (P2, P6) (Table 2) revealed a general decrease in respiratory chain enzyme activities. An oxidation of NADH and succinate and cytochrome c oxidase respiration decreased to 20%–55% of the controls and activities of respiratory complexes I+III, II+III, and IV decreased to 15%–81%, respectively, indicating more extensive impairment in P2 heart ventricles (Table 2). The impairment of succinate respiration was the most profound with a mean of 277 pmol O_2_/s/mg. This was much lower than in our historical controls from donor hearts unsuitable for transplantation (653 ± 244 pmol O_2_/s/mg, *n* = 38) and even myocardium explanted during heart transplantation or ventricular assist device implantation (508 ± 211 pmol O_2_/s/mg, *n* = 91) [25]. Western blot quantification of mitochondrial proteins showed a decrease in specific content of respiratory chain complexes, also more pronounced in P2, where a very low content of complexes IV and I was associated with the upregulation of complex II (Figure 5B). Other mitochondrial proteins, as porin (shown in Figure 5A) and adenine nucleotide translocator (not shown) were less affected. Analysis of native forms of respiratory chain complexes by BlueNative electrophoresis (Figure 5C) confirmed a marked reduction of complexes I and IV in P2 heart and further showed that it led to a pronounced decrease of high molecular weight respiratory supercomplexes consisting of complexes I, III, and IV. Similar, yet a smaller decrease of supercomplexes was observed in soleus of R349P desmin knock-in mouse [15] or heart of desmin knockout mouse [27]. The content of mitochondrial DNA (relative to nuclear DNA, D-loop/GAPDH, and 16S RNA/GAPDH) was slightly decreased in P6 heart ventricles (60%–90% of the average value of the controls) but was unchanged in P2 heart. These data indicate mild to pronounced attenuation of the energetic function of mitochondria due to a decreased content and activity of respiratory complexes and supercomplexes in failing hearts of patients with desminopathy.

**Figure 6:**
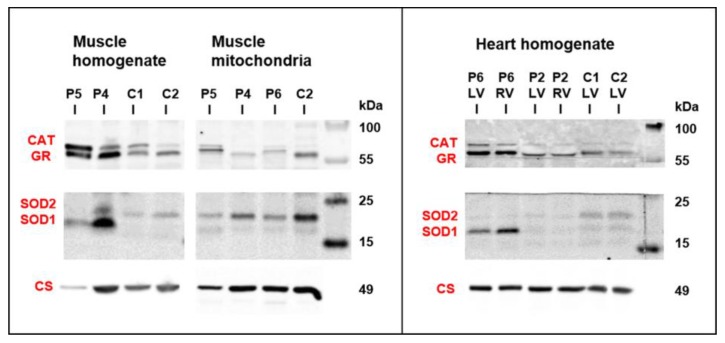
Western blot detection of antioxidative enzymes in skeletal muscle and heart. Both P4 and P5 muscle homogenates revealed variable increase in antioxidative enzymes glutathione reductase (GR), catalase (CAT), and superoxide dismutases 1 and 2 (SOD1, SOD2). Increased content of CAT, GR, and SOD1 was also found in heart ventricles of P6. Protein aliquots–muscle homogenate 30 µg, muscle mitochondria 15 µg, and heart homogenate 20 µg. For comparison, citrate synthase signal (CS) from Figure 5 is shown.

As changes in mitochondria energetic function can affect a generation of reactive oxygen species, the content of antioxidative enzymes was analyzed by Western blot analysis in skeletal muscle and heart samples from patients with desminopathy (Figure 6). Both P4 and P5 muscle homogenates revealed highly increased glutathione reductase (GR) and superoxide dismutase 1 (SOD1) as well as a variable increase of catalase (CAT) and superoxide dismutase 2 (SOD2). Higher CAT was found in P5 isolated mitochondria, while the most increased SOD1 was of extra-mitochondrial origin (also apparent from the SOD1/SOD2 ratio). An increased content of GR and SOD1 was also found in heart ventricles of P6.

## 4. Discussion

Firstly, the prevalence of desminopathy in a large cohort of patients with an unexplained etiology of cardiomyopathy assessed with WES was 1.8%. Secondly, the presence of pathological desmin aggregates in myocardial/skeletal muscle samples of P1–P3 and decreased myocardial desmin expression in P4 suggested a pathogenicity of two novel *DES* variants and two *DES* variants previously classified as of uncertain significance. Thirdly, a pathogenicity of one variant of uncertain significance (DES-p.(K43E)) was supported also by abnormal desmin filament formation and its cytoplasmic aggregation in transfected HT1080 cells and in iPSC-derived cardiomyocytes. Fourthly, we provided further evidence for LVNC as a novel phenotype of desminopathy. Fifthly, we described secondary mitochondrial dysfunction in skeletal muscle and in myocardium, which was in case of myocardial succinate respiration more profound than in end-stage heart failure of other etiology. To the best of our knowledge, this seems to be the first comprehensive description of mitochondrial dysfunction in human myocardium affected by desminopathy. Finally, secondary mitochondrial dysfunction and/or an extensive left ventricular late gadolinium enhancement in desminopathy may imitate a primary mitochondrial disease or an inflammatory cardiomyopathy.

### 4.1. Clinical and Histopathological Correlates of Desminopathy

The majority of 68 pathogenic desmin variants that were reported so far are missense or small in-frame deletion variants localized in the helical rod domain [26,28]. A phenotype-genotype correlation meta-analyses revealed that pathogenic variants in the rod 2B domain of *DES* are common among patients with both skeletal and cardiac muscle phenotype, whereas head and tail domain pathogenic variants result mainly in clinically isolated cardiac phenotype [1,29,30]. In agreement with these findings, we found that two *DES* variants in head region (p.(K43E), p.(S57L)) and one novel *DES* variant (p.(A210D)) in the 1B helical domain had in probands isolated cardiac involvement. On the other hand, the novel *DES* mutation located in the 2B helical domain (p.(Q364H)) and two known *DES* variants (p.(R406W), p.(R454W)) affected both the cardiac and skeletal muscle.

Immunohistochemistry revealed pathological desmin aggregates in skeletal or cardiac myocytes in five probands from our study group. Importantly, desmin aggregates were absent in the deltoid muscle of proband 6 (*DES*-p.(R454W)), but present in her myocardial samples. Desmin aggregates were completely absent in proband 4 (*DES*-p.(Q364H)). Immunohistochemistry and electron microscopy of diagnostic muscle soleus biopsy and post-mortem myocardial and intercostal muscle samples failed to detect any pathological protein aggregates. Interestingly, Western blot analysis of the myocardial sample showed a decreased expression of desmin suggesting decreased protein synthesis. This is in agreement with the experience of pathologists that myopathological findings in genetically proven desminopathies may range from no overt pathology over subtle myopathic changes with sporadic protein aggregates to the picture of a vacuolar myopathy [31]. An absence of desmin aggregates has been recently documented both in autosomal dominant [32] and autosomal recessive [33] desminopathy.

### 4.2. Novel Cardiac Phenotypes of Desminopathy

In addition to known cardiac phenotypes of desminopathy like DCM, RCM HCM, and ACM [1,28,29], we observed LVNC as a relatively novel phenotype. So far, *DES* variants have been associated with LVNC just in a few individuals [34,35,36,37]. The first report from Arbustini et al. [34] described a family with a segregation of *DES* variant p.(G84S) with non-obstructive hypertrophic cardiomyopathy and one case of LVNC. Another group [35] reported one sporadic case of LVNC in a child with a *DES* variant p.(L398P). An occurrence of two cases of LVNC in one family has been recently associated with an in-frame mutation of desmin p.(Q113_L115del) affecting the α–helical rod domain [36] with a formation of typical desmin-immunoreactive aggregates. We expanded the available evidence by a description of another familial occurrence of two cases of LVNC associated with the desmin variant p.(Q364H) with a decreased myocardial expression of desmin and absent desmin aggregates in myocardial/skeletal biopsy.

Recently, a novel phenotype of desminopathy describing left ventricular arrhythmogenic cardiomyopathy was reported to have a significant amount of subepicardial fibrosis [32]. A similar phenotype had our P2, which mimicked inflammatory cardiomyopathy by an extensive late gadolinium enhancement in the left ventricle and persistent elevation of cardiac troponins. However, ventricular ectopy and an inversion of T waves in inferior and precordial leads were absent. Taken together, the presence of desminopathy should be considered also in unexplained cases of LVNC and non-ischemic left ventricular systolic dysfunction with an extensive subepicardial or intramural fibrosis.

### 4.3. Mitochondrial Dysfunction in Desminopathy

Desminopathy may also imitate a mitochondrial disease, as was shown by Mc Cormic et al. [16]. The presence of SDH positive/COX negative muscle fibers, decreased activities of mitochondrial respiratory chain enzymes, and reduced mitochondrial DNA content in skeletal muscle biopsy lead to the suspicion of mitochondrial disease. We observed similar findings in our patient (P6) with an absence of desmin aggregates and signs of mitochondrial dysfunction in deltoid muscle biopsy. The correct diagnosis in our case provided genetic testing and immunostaining of myocardial samples.

Studies in desmin null mice and patients with recessive desmin-null muscular dystrophy revealed abnormalities in nuclear and mitochondrial localization and morphology, as well as impaired mitochondrial respiratory capacity [13,14]. Secondary mitochondrial dysfunction was also confirmed by Schröder et al. [38] and Vincent et al. [39] in skeletal muscle biopsies of heterozygous patients with desminopathy. Furthermore, Vincent et al. [39] reported a deficiency of respiratory chain complex I and IV compared to age matched controls and a low mitochondrial mass compared to controls. Our morphological and functional data from skeletal muscle samples are in agreement with the above mentioned studies and further evidence [40,41]. We observed a variable mitochondrial dysfunction characterized by a decreased expression of mitochondrial respiratory chain components and other mitochondrial proteins, as well as decreased enzyme activities, suggesting secondary changes in mitochondrial energetic function. The upregulation of several anti-oxidative enzymes, in particular that of superoxide dismutase 1 in homogenates, but not in isolated mitochondria, indicated increased antioxidative defense outside mitochondria.

Novel findings provided our analysis of myocardial energetic function in the explanted failing hearts of two probands with desminopathy. In one case harboring p.(R454W) desmin tail mutation we found a mild decrease in the content and activities of respiratory chain complexes while in the other case with p.(S57L) mutation in the desmin head region was present a very pronounced decrease in mitochondria proteins and an alteration of the bioenergetics function. Interestingly, changes in respiratory chain enzymes thus also caused a downregulation of respiratory supercomplexes that are expected to modulate the catalytic function as well as reactive oxygen species production by the respiratory chain [42]. This was associated with a decrease in several marker proteins of different mitochondrial compartments suggesting a complex mitochondrial dysfunction. The most pronounced was the impairment of myocardial succinate respiration, which was in our patients with desminopathy more profound than in end-stage heart failure of other etiologies.

### 4.4. Study Limitations

There are several limitations to our study. First, the small study group size reflects the rare occurrence of desminopathy and may limit the general applicability of the study results. Secondly, the small size of affected families limited the segregation studies. However, WES enabled us to exclude the presence of other pathogenic variants in cardiomyopathy- and skeletal myopathy-related genes, including genes coding mitochondrial proteins. Thirdly, an assessment of the mitochondrial function in tissues was possible only in a subgroup of patients with a clinical indication to biopsy or undergoing cardiac surgery. Finally, decreased desmin expression in P4 with a missense variant of *DES* might be related to replacement fibrosis of the myocardium or epigenetic factors. Unfortunately, we cannot provide data supporting any of these hypotheses.

## 5. Conclusions

Desminopathy is a rare cause of cardiomyopathy and/or skeletal muscle myopathy with a pleomorphic clinical presentation and poor prognosis. This diagnosis should also be considered in individuals with LVNC. Differential diagnosis also includes mitochondrial and inflammatory myocardial diseases.

## Figures and Tables

**Table:** **(A)**

Enzyme Activity of Muscle Homogenates (nmol/min/mg protein)	P4	P5	P6	Controls *n* = 30
Complex IV	130.1	38.1	81.8	68–213
Citrate synthase (CS)	109.5	41.4	97.8	48–128
Complex IV/CS	1.19	0.92	0.84	080–160
Coenzyme Q10 content (pmol/mg)	282.9	140.5	112.5	180–460

**Table:** **(B)**

Respiratory Activity of Permeabilized Muscle Fibers (pmol O_2_/s/mg protein)	P6	Controls *n* = 9
ADP-stimulated oxidation of NADH-dependent substrates	7.4	16–26
ADP-stimulated oxidation of succinate	10.7	9–18
Cytochrome *c* oxidase respiration	63	43–83

**Table:** **(C)**

Enzyme Activity of Isolated Mitochondria (nmol/min/mg protein)	P4	P5	P6	Controls *n* = 30
Complex I	328.5	230.8	131.2	110–290
Complex I+III	94.1	18.7	53.2	126–316
Complex II	69.7	50.5	49.5	21–93
Complex II+III	174.2	92.9	146.7	82–251
Complex III	303.0	342.7	535.0	200–600
Complex IV	578.4	311.6	236.6	658–1552
Citrate synthase	372.5	240.4	384.2	435–1234
Complex I/CS	0.88	0.96	0.34	0.17–0.41
Complex I+III/CS	0.25	0.07	0.13	0.07–0.27
Complex II/CS	0.19	0.21	0.13	0.04–0.12
Complex II+III/CS	0.47	0.39	0.38	0.35–0.36
Complex III/CS	0.81	1.43	1.39	0.56–1.46
Complex IV/CS	1.55	1.30	0.62	0.82–1.88

Abbreviations: ADP–adenosine diphosphate, ATP–adenosine triphosphate, and NADH–reduced form of nicotinamide adenine dinucleotide.

**Table 2:** Activities of respiratory chain enzymes and mtDNA content in hearts of proband 2 and 6.

Respiratory/Enzyme Activity	P2 Left Ventricle	P2 Right Ventricle	P6 Left Ventricle	P6 Right Ventricle	Controls *n* = 38
(pmol O_2_/s/mg) NADH respiration	245	203	448	229	235–2356
Succinate respiration	295	242	254	319	365–1529
Cytochrome *c* oxidase respiration	766	991	1003	1047	561–4120
(nmol/min/mg) Complex I+III	26.1	30.3	145.1	83.9	44–386
Complex II+III	88.0	71.3	59.5	56.7	27–195
Complex IV	262.6	432.4	430.5	276.8	389–1989
Citrate synthase (CS)	915.9	975.2	562.2	483.6	446–1207
(activity ratio)					
Complex I+III/CS	0.03	0.03	0.26	0.17	0.09–0.63
Complex II+III/CS	0.10	0.07	0.11	0.12	0.04–0.37
Complex IV/CS	0.29	0.44	0.77	0.57	0.54–2.60
mtDNA content					
(2^−ΔCt^)					
D-loop/GAPDH	4980	5499	2863	3592	2052–10519
16S RNA/GAPDH	11629	10914	8017	7299	3715–15843

Enzyme activities are expressed per mg protein, mtDNA content is expressed as 2−ΔCt value indicating the number of mtDNA copies per a haploid genome. Abbreviation: GADPH–glyceraldehyde 3-phosphate dehydrogenase.

## Supplementary Materials

The following are available online at https://www.mdpi.com/2077-0383/9/4/937/s1, Table S1 Primers for PCR Amplification of DES for segregation analysis in families, Table S2 Main clinical characteristics of probands, Table S3 Additional clinical, electrocardiographic, laboratory and echocardiographic data of probands, Table S4 Rare variants of non-desmin genes in probands, frequency in Exac database less than 0.00001.

## Abbreviations and Acronyms

ACM	arrhythmogenic cardiomyopathy
CAT	catalase
COX	cytochrome c oxidase
DCM	dilated cardiomyopathy
DES	desmin gene
GADPH	glyceraldehyde 3-phosphate dehydrogenase
GR	glutathione reductase
HCM	hypertrophic cardiomyopathy
LVNC	left ventricular non-compaction cardiomyopathy
NADH	reduced form of nicotinamide adenine dinucleotide
MPS	massively parallel sequencing
RCM	restrictive cardiomyopathy
SDH	succinate dehydrogenase
SOD	superoxide dismutase
WES	whole exome sequencing
