# Supplementary material for: Desminopathy: Novel Desmin Variants, a New Cardiac Phenotype, and Further Evidence for Secondary Mitochondrial Dysfunction
Source: J Clin Med. 2020 Mar 29;9(4):937. doi: 10.3390/jcm9040937 (PMC7231262; doi:10.3390/jcm9040937)
Supplement: Supplementary file 1 [file jcm-09-00937-s001.pdf]

SUPPLEMENTARY MATERIALS

SUPPLEMENTARY METHODS:

Molecular-genetic analysis

Genomic DNA of all available individuals was extracted from peripheral blood leukocytes using a standard technology. WES was performed on 1 μg of DNA in all individuals tested. For DNA enrichment, barcoded DNA libraries and SeqCap EZ MedExome Target Enrichment Kit (SeqCap EZ MedExome Probes used with mitochondrial genome design, Roche, Madison, USA) were used according to the manufacturer's protocol. MPS was performed on the captured barcoded DNA library using the Illumina Hiseq 2500 system at the genomic facility at the National Coordination Centre for Rare Diseases (www.nkcvo.cz) at Motol University Hospital; Prague. The resulting FASTQ files were aligned to the Human Genome Reference (hg19) using Novoalign software (ver. 3.02.10; www.novocraft.com). Following genome alignment, conversion of SAM format to BAM and duplicate removal were performed using Picard Tools (ver. 1.129; broadinstitute.github.io/picard/). The Genome Analysis Toolkit, GATK (3.3) **[43]** was used for local realignment around indels, base recalibration and variant recalibration and genotyping. Variant annotation was performed with SnpEff **[44]** and GEMINI **[45]**. Rare variants were defined as having a frequency ≤ 0.05% among control samples. Comparisons were made with ethnically matched controls available from the database of genomics variants maintained by the Czech National Center for Medical Genomics; (http://ncmg.cz/en) and the publicly available genotype data from subjects of European origin sequenced in the 1000 Genomes Project, Exome Aggregation Consortium (ExAC) and The Genome Aggregation Database (gnomAD) **[46]**. Identified genetic variants were filtered according to the expected autosomal dominant model of disease inheritance and evaluated according to the biological relevance of corresponding candidate genes. Candidate variants were visualized in Integrative Genomics Viewer (IGV; ver. 2.3.32) **[47].**

Immunohistochemical detection of desmin and electron microscopy

Immunohistochemistry was performed on both skeletal muscle and myocardium samples according to standard protocols to study desmin expression with the anti-Desmin antibody (mouse monoclonal antibody, clone D33 diluted 1:200; Agilent pathology solutions-Dako, USA). The antigen-antibody complexes were visualized by biotin-streptavidin detection systems (ChemMate Detection kit, Agilent pathology solutions-Dako, USA). Western blot was used to assess quantity of desmin in the myocardial samples of P2, P4 and P6. Frozen tissue was processed in a Teflon/glass homogenizer in SB Buffer (50 mM Tris pH 6.8, 50 mM DTT, 2% SDS 1xPIC), incubated for 1 hour at 37 °C, sonicated on ice and centrifuged for 5 min at 18 000g to remove non-lysed proteins. Denatured protein samples (30 µg aliquots) were separated on 10% SDS–PAGE and blotted onto a PVDF membrane. DES was visualized by incubation with monoclonal mouse anti-human desmin (Agilent pathology solutions-Dako, USA) followed by incubation with anti-mouse IgG-peroxidase antibody A8924 (Merck, USA), and detection by ClarityTM Western ECL Substrate (Bio-Rad, USA). Mitochondrial ATP synthase subunit β (ATP5B) was detected with mouse monoclonal anti-ATP5B antibody ab14730 (Abcam, USA).

For electron microscopy, specimens (approx. 1 mm^3) were fixed in a glutaraldehyde solution, diluted in 2% with sodium cacodylate buffer, pH 7.4 for 6 hours at 4°C, washed in the same buffer, post-fixed for 2 hours in 1% osmium tetroxide, dehydrated through a graded acetone series and embedded in epoxy resin. Semi-thin sections (1.5 µm) were mounted on acid-cleaned slides and stained with 1% toluidine blue. Ultrathin sections were double stained with uranyl acetate and lead citrate. The grids were examined at 80 kV with transmission electron microscope.

Analysis of mitochondrial function in biopsies

Protein expression was analyzed by Western Blotting as described previously **[48]**. Homogenates and mitochondria were denatured for 20 min at 40 ˚C in 2 % (v/v) mercaptoethanol, 4 % SDS (w/v), 10 mM Tris-HCl and 10 % (v/v) glycerol and proteins resolved by SDS-PAGE **[49]** were electro-transferred to a PVDF membrane. Specific primary antibodies were used to assess respiratory chain enzymes (NDUFA9 subunit of complex I, SDHA subunit of complex II, Core2 subunit of complex III, Cox4 subunit of complex IV, F_1-subunit of complex V), mitochondrial proteins citrate synthase, adenine nucleotide translocator, phosphate translocator, porin, and antioxidative enzymes - superoxide dismutases 1 and 2, catalase, glutathione reductase. For quantitative detection, infra-red fluorescent secondary antibodies were used and the signal was quantified using Odyssey Infrared Imager (LI-COR Biosciense s) and AIDA 3.21 Image Analyzer software (Raytest). Specific content of each protein antigen was expressed in AU/mg protein and the content in proband samples was calculated in percent of the mean value of the controls. In case of heart samples, the controls (hearts of organ donors that were not used for transplantation for technical or medical reasons) obtained in a previous study **[25]** were used.

Mitochondrial DNA content. Genomic DNA was isolated and quantitative real-time PCR of two mitochondrial target sequences (16S and D-loop) and one nuclear target glyceraldehyde 3-phosphate dehydrogenase (GAPDH) was performed to quantify mitochondrial DNA (mtDNA) content relative to nuclear DNA content (16S/GAPDH and D-loop/GAPDH ratios) **[50].** The mtDNA content was calculated from threshold cycle (Ct) values as $2^{-\Delta Ct}$ where $\Delta Ct = Ct(mtDNA) – Ct(GAPDH)$. The $2^{-\Delta Ct}$ value indicates the number of mtDNA copies per a haploid genome.

Activities of respiratory chain complexes and citrate synthase. The activities of the mitochondrial enzymes NADH: ubiquinone oxidoreductase (complex I), succinate: coenzyme Q reductase (complex II), ubiquinol: cytochrome c oxidoreductase (complex III), cytochrome c oxidase (complex IV), NADH: cytochrome c reductase (complex I+III), succinate: cytochrome c reductase (complex II+III) were measured spectrophotometrically at 37 °C in isolated mitochondria according to Rustin et al. **[51]** and citrate synthase according to Srere **[52]**. Protein concentration was measured according to Lowry et al. **[53]**.

High resolution oxygraphy.

Succinate respiration, NADH (reduced form of nicotinamide adenine dinucleotide) respiration and cytochrome *c* oxidase respiration were assessed in heart homogenates and muscle fibers using the Oxygraph-2k (OROBOROS Instruments) **[48]**. Muscle fibers were separated mechanically according to Kunz et al. **[54]**, and oxygen consumption by saponin-skinned muscle fibers was determined using multiple substrate inhibitor titrations as described previously **[55].**

Content of coenzyme Q10.

Total Q10 content was determined in muscle homogenate by HPLC with UV detection at 275nm according to Mosca et al. **[56]**. Results were expressed as pmol of Q10/ mg of protein.

Supplementary references:

43. McKenna, A; Hanna, M.; Banks, E.; Sivachenko, A.; Cibulskis, K.; Kernytsky, A.; Garimella, K.; Altshuler, D.; Gabriel, S.; Daly, M.; et al. The Genome Analysis Toolkit: a MapReduce framework for analyzing next-generation DNA sequencing data. Genome Res. **2010**, 20, 1297–1303, doi:

10.1101/gr.107524.110.

44. Cingolani, P.; Platts, A.; Wang le, L.; Coon, M.; Nguyen, T.; Wang, L.; Land, S.J.; Lu, X.; Ruden, D.M. A program for annotating and predicting the effects of single nucleotide polymorphisms, SnpEff: SNPs in the genome of Drosophila melanogaster strain w1118; iso-2; iso-3. Fly (Austin) **2012**, 6, 80-92, doi: 10.4161/fly.19695.

45. Paila, U.; Chapman, B.A.; Kirchner, R.; Quinlan, A.R.; GEMINI: integrative exploration of genetic variation and genome annotations. PLoS Comput Biol. **2013**, 9, e1003153, doi: 10.1371/journal.pcbi.1003153.

46. Lek, M.; Karczewski, K.J.; Minikel, E.V.; Samocha, K.E.; Banks, E.; Fennell, T.; O'Donnell-Luria, A.H.; Ware, J.S.; Hill, A.J.; Cummings, B.B.; et al. Exome Aggregation Consortium. Analysis of protein-coding genetic variation in 60,706 humans. Nature **2016**, 536, 285-291, doi: 10.1038/nature19057.

47. Thorvaldsdóttir, H.; Robinson, J.T.; Mesirov, J.P. Integrative Genomics Viewer (IGV): high-performance genomics data visualization and exploration. Brief Bioinformatics **2013**, 14, 178–192, doi: 10.1093/bib/bbs017.

48. Pecinova, A.; Drahota, Z.; Nuskova, H.; Pecina, P.; Houstek, J. Evaluation of basic mitochondrial functions using rat tissue homogenates. Mitochondrion **2011**, 11, 722-728, doi: 10.1016/j.mito.2011.05.006.

49. Schagger, H.; von Jagow, G. Tricine-sodium dodecyl sulfate-polyacrylamide gel electrophoresis for the separation of proteins in the range from 1 to 100 kDa. Anal Biochem **1987**, 166, 368-379, doi: 10.1016/0003-2697(87)90587-2.

50. Havlickova Karbanova, V.; Cizkova Vrbacka, A.; Hejzlarova, K.; Nuskova, H.; Stranecky, V.; Potocka, A.; Potocka, A.; Kmoch, S.; Houstek, J. Compensatory upregulation of respiratory chain complexes iii and iv in isolated deficiency of atp synthase due to tmem70

mutation. Biochimica et biophysica acta. **2012**, 1817, 1037-1043, doi: 10.1016/j.bbabio.2012.03.004.

51. Rustin, P.; Chretien, D.; Bourgeron, T.; Gérard, B.; Rötig, A.; Saudubray, J.M.; Munnich, A. Biochemical and molecular investigations in respiratory chain deficiencies. Clin Chim Acta **1994**, 228, 35-51, doi: 10.1016/0009-8981(94)90055-8.

52. Srere, P.A.; John, M.L. Citrate synthase : [EC 4.1.3.7. Citrate oxaloacetate-lyase (CoA-acetylating)]. Methods in Enzymology **1969**, Academic Press, Volume 13, 3-11.

53. Lowry, O.H.; Rosebrough, N.J.; Farr, A.L.; Randall, R.J. Protein measurement with the Folin phenol reagent. J Biol Chem **1951**, 193, 265-275.

54. Kunz, W.S.; Kuznetsov, A.V.; Schulze, W.; Eichhorn, K.; Schild, L.; Striggow, F.; Bohnensack, R.; Neuhof, S.; Grasshoff, H.; Neumann, H.W.; et al. Functional characterization of mitochondrial oxidative phosphorylation in saponin-skinned human muscle fibers. Biochim Biophys Acta **1993**, 1144, 46–53, doi: 10.1016/0005-2728(93)90029-f.

55. Wenchich, L.; Drahota, Z.; Honzik, T.; Hansikova, H.; Tesarova, M.; Zeman, J.; Houstek, J. Polarographic evaluation of mitochondrial enzymes activity in isolated mitochondria and in permeabilized human muscle cells with inherited mitochondrial defects, Physiol Res **2003,** 52, 781–788.

56. Mosca, F.; Fattorini, D.; Bompadre, S.; Littarru, G.P. Assay of Coenzyme Q10 in plasma by a single dilution step. Anal Biochem. **2002**, 305, 49-54, doi:10.1006/abio.2002.5653.

SUPPLEMENTARY TABLES:

Table S1: Primers for PCR Amplification of ***DES*** for segregation analysis in families

Name	Sequence 5´- 3´	Genomic Position	Family
DES_F1_U	GGCTGATGTCAGGAGGGATAC	2: 220283052 220283072	F1, F2
DES_F1_L	CAGAAAGAGGAGTCTGGGGTG	2: 220283775 220283795	F1, F2
DES_F3_U	CTGTCTCTCCTGCCTCTACCCA	2: 220284679 220284700	F3
DES_F3_L	GCGATCTCCTCGTTGAGAGATTCA	2: 220285020 220285043	F33
DES_F4_U	AGCTTTCTTTGGGCTGCTAGT	2: 220285999-220286019	F4, F5
DES_F4_L	AGGTGGCCTTGGTTAATTCTC	2: 220286731 220286751	F4, F5
DES_F6_U	AACAAGCCTGTCTTGAGGGG	2: 220290254 220290273	F6
DES_F6_L	AGGAAGAGAAGCCCAGTCCA	2: 220290645 220290664	F6

Table S2: Main clinical characteristics of probands

	**Proband 1**	**Proband 2**	**Proband 3**	**Proband 4**	**Proband 5**	**Proband 6**
Mutation of desmin	p.K43E	p.S57L	p.A210D	p.Q364H	p.R406W	p.R454W
Age of the first examination (years)	17	25	47	47	25	45
Gender	Male	Female	Male	Male	Female	Female
Results of family screening	Negative	Father of the proband, trifascicular block, mild elevation of creatinine phosphokinase.	Sister diagnosed with incipient DCM at 52 years (EF 51%, LVEDD 60mm).	Mother died of heart failure at 58years. Daughter of the proband-noncompacted myocardium in the apical inferolateral third of the LV (ratio 5:1 to compacted layer), preserved systolic function.	Negative	Mother had cardiomyopathy, AV blockade, myopathy, died 43 years old
Type of cardiomyopathy	Biventricular form of arrhythmogenic cardiomyopathy	DCM	DCM	LVNC	Right ventricular form of arrhythmogenic cardiomyopathy	Restrictive
Complete atrioventricular block	No	No	No	No	Yes, dual chamber ICD (25 yrs)	Yes, dual chamber pacemaker (21 yrs)
Sustained VT/ resuscitated cardiac arrest	Yes (12 yrs)	Yes (27 yrs)	No	No	No	No
Myocardial biopsy, type of samples	Explanted heart at transplantation	Endomyocardial biopsy and explanted heart at transplantation	Endomyocardial biopsy	Explanted heart post-mortem	-	Explanted heart at transplantation

Abnormal desmin distribution and formation of desmin-positive aggregates in cardiomyocytes by IH	Positive	Positive	Positive	Negative	-	Positive
Pathological dense granulofilamentous inclusions in the cytoplasm of cardiomyocytes by ELMI	Positive	Positive	-	Negative	-	Positive
The first episode of heart failure (years)	21	25	47	48	39	45
Weakness/atrophy upper limbs	No	Very slight proximal weakness	No	Marked atrophy of muscles	Mild proximal and slight distal weakness	Slight proximal atrophy
Weakness/atrophy lower limbs	No	Slight proximal weakness	No	Marked atrophy of muscles	Mild proximal weakness	Slight proximal atrophy
Bulbar symptoms	No	No	No	Mild bulbar speech since 62 yrs	Mild bulbar speech since 39 yrs	Mild bulbar speech since 44 yrs
Vital capacity (L/%)	3,91l (81%)	2.67l (67%)	-	3,75 (75%)	2,2 (60%)	2,37 (71%)
Electromyography	Slightly myogenic	Normal in muscles, incipient sensoric polyneuropathy	Normal in muscles, sensoric polyneuropathy	Myogenic	Myogenic	Myogenic
Creatinine phosphokinase (μkat/l) (range 0.5-3.33)	0,54	0,88	3,38	2,01	7,22	1,57
Skeletal biopsy, type of specimen	-	-	-	Soleus muscle biopsy + post-mortem intercostal muscles	Solueus muscle	Deltoid muscle
Detection of desmin-positive aggregates	-	-	-	Negative	Positive	Negative

in skeletal myocytes by IH						
Pathological dense granulofilamentous inclusions in the cytoplasm of skeletal myocytes by ELMI	-	-	-	Negative	Positive	Positive
Significant clinical events	Heart transplant (23y)	Heart transplant (28y)	-	Died of terminal heart failure, cachexia (64y)	Aphagia, aspiration pneumonia, died of heart failure (40 y)	Heart transplant (46y)

Abbreviations: ELMI= electron microscopy, DCM= dilated cardiomyopathy, ICD= implantable cardioverter-defibrillator, IH= immunohistochemistry, LV= left ventricle, LVEDD= left-ventricular enddiastolic diameter, LVEF= left ventricular ejection fraction, LVNC= left-ventricular noncompaction cardiomyopathy, VT= ventricular tachyarrhythmia

Table: S3: Additional clinical, electrocardiographic, laboratory and echocardiographic data of probands

Variable (units)<br>Reference range	**Proband 1**		**Proband 2**		**Proband 3**		**Proband 4**		**Proband 5**		**Proband 6**	
Duration of follow-up (months)	78		36		34		180		188		21	
	BL	Last FU	BL	Last FU	BL	Last FU	BL	Last FU	BL	Last FU	BL	Last FU
Age (years)	17	23	25	28	47	50	47	63	25	40	45	50
NYHA class	I	IV	III	III	IV	III	II	IV	II	III	III-IV	IV
Rhythm	Sinus rhytm	Sinus rhythm	Sinus rhytm	Sinus rhytm	Sinus rhythm	Sinus rhythm	Sinus rhytm	Sinus rhytm	Sinus rhytm, III. degree AV block intermit.	AV sequential pacing	AV sequential biventricular stimulation	AV sequential biventricular stimulation
PQ interval (ms) (120-200)	174	198	162	176	170	180	162	164	220	-	-	-
QRS duration (ms) (80-110)	100	104	100	114	120	130	98	114	130	122	130	156

Serum creatinine (µmol/l)<br>(60-106)	73	87,2	92	92,1	82	87	-	104	56,2	43,4	63,8	92
B-type natriuretic peptide (ng/l)<br>(10-73)	-	1133	621	1374	307	558	-	3403	-	735,6	1137,5	2308
Cardiac troponins<br>(I 0-0.03 µg/l,<br>T 0-14 ng/l)	Troponin I <0.03	Hs TNT<br>10,71	Hs TNT<br>168,3	Hs TNT<br>318,6	-	-	-	Hs TNT<br>25,02	Troponin I 0,13	-	Hs TNT<br>37,21	Hs TNT<br>34,95
LVEDD (mm)<br>(males 42-59, females 39-53)	51	59	60	66	70	73	64	72	47	53	47	55
Left ventricular ejection fraction (%)<br>(55-70)	60	20-25	26	20-25	16	22	25	24	55-60	45-50	30-35	20-25
Interventricular septum (mm)<br>(males 6-10, females 6-9)	9	10	8	8	8	10	9	7	12	7	8	7

Posterior wall (mm)<br><br>(males 6-10, females 6-9)	8	7	10	10	8	9	9	6	11	7	8	6
E/A ratio	1,8	0,8	4,1	-	2,21	2,27	-	3,0	-	1,9	-	-
E/Em ratio<br><br>(<8)	-	6,5	12	11,8	8,7	8,5	-	20,6	-	15	10,9	9,6
Restrictive mitral pattern	no	No	Yes	No	yes	yes	-	Yes	-	no	No	Yes
Mitral regurgitation (scale) (1-4)	1/4	1/4	3/4	2-3/4 (Mitraclip)	2/4	2/4	2/4	3-4/4	1/4 max	1/4	2/4	2/4
Left atrium diameter (mm)<br><br>(27-38)	32	29	46	47	51	57	39	46	35	43	46	46
LAVI (cm3/m2)<br><br>(16-28)	-	20,9	60,9	57,1	53	50	-	104,4	-	46,5	70,4	78,2
RVD1 (mm)<br><br>(20-28)	-	55	31	35	27	25	-	52	42	46	27	46
Right ventricular systolic	None	Moderate to severe	Moderate	None	None	None	None	Moderate	Mild	Moderate to severe	Severe	Moderate to severe

dysfunction (scale)												
Right ventricular ejection fraction (%)	Normal	TAPSE 14 mm,<br>TDI 7 cm/s	TAPSE 18 mm, TDI 9 cm/s	TAPSE 18 mm, TDI 10 cm/s	- (TAPSE 22mm)	- (TAPSE 19mm)	-	TAPSE 11 mm, TDI 6 cm/s	-	TAPSE 11 mm,<br>TDI 9 cm/s	TDI 6 cm/s	TAPSE 7 mm, TDI 6 cm/s
Tricuspidal regurgitation (scale)	1/4	¾	1/4	2/4	1/4	1	1/4 max	2/4	1/4 max	2/4	2/4	1/4

Abbreviations: Hs TNT= high sensitivity troponin T, LAVI= left atrium volume index, NYHA= New York Heart Association functional class, RVD1= right ventricular diastolic diameter

Table S4: Rare variants of non-desmin genes in probands, frequency in Exac database less than 0,00001.

Proband 1

Chr	Position	Ref	Alt	Gene	Accesion	cDNA change	AAA change
1	984378	G	A	AGRN	NM_198576.3	c.4237G>A	G1413S
1	94520771	G	C	ABCA4	NM_000350.2	c.2483C>G	P828R
1	156874735	G	A	PEAR1	NM_001080471.1	c.206+91G>A	
4	861088	G	T	GAK	NM_005255.3	c.2528C>A	P843H
5	92920919	C	G	NR2F1	NM_005654.5	c.190C>G	P64A
5	171318519	C	G	FBXW11	NM_012300.2	c.741G>C	Q247H
6	153077337	C	A	VIP	NM_003381.3	c.404C>A	T135N
6	160494990	A	G	IGF2R	NM_000876.3	c.5149A>G	I1717V
8	22052098	T	G	BMP1	NM_006129.4	c.1438T>G	F480V
11	59611527	G	T	GIF	NM_005142.2	c.81C>A	S27=
11	75111821	G	A	RPS3	NM_001260506.1	c.114G>A	E38=
11	123597390	C	T	ZNF202	NM_003455.3	c.1262G>A	G421E
12	18889224	T	G	PLCZ1	NM_033123.3	c.66A>C	E22D
12	49431411	C	G	KMT2D	NM_003482.3	c.9728G>C	S3243T
18	3193959	G	A	MYOM1	NM_003803.3	c.291-3C>T	

Proband 2

Chr	Position	Ref	Alt	Gene	Accesion	cDNA change	AAA change
1	32852373	T	C	BSDC1	NM_001143888.2	c.189+7A>G	
1	109547295	G	C	WDR47	NM_001142550.1	c.1197C>G	p.Ile399Met
1	111690658	C	A	CEPT1	NM_001007794.2	c.322C>A	p.Pro108Thr
1	152080501	A	T	TCHH	NM_007113.3	c.5192T>A	p.Leu1731Gln
1	152325588	A	T	FLG2	NM_001014342.2	c.4674T>A	p.His1558Gln
2	27466112	A	G	CAD	NM_004341.4	c.6527A>G	p.Lys2176Arg
2	217005962	A	G	XRCC5	NM_021141.3	c.1396A>G	p.Lys466Glu

2	223559960	CA	C	MOGAT1	NM_058165.2	c.806delA	p.Gln269ArgfsTer6
3	100499018	T	C	ABI3BP	NM_015429.3	c.2065A>G	p.Arg689Gly
4	47951911	C	G	CNGA1	NM_001142564.1	c.445G>C	p.Glu149Gln
4	129083409	A	G	LARP1B	NM_018078.3	c.1585A>G	p.Thr529Ala
5	132161121	G	A	SHROOM1	NM_133456.2	c.712C>T	p.Pro238Ser
6	32094036	G	T	ATF6B	NM_004381.4	c.343-7C>A	
6	44900430	C	T	SUPT3H	NM_003599.3	c.872G>A	p.Arg291Gln
6	78471065	A	G	MEI4	NM_001282136.2	c.451A>G	p.Met151Val
6	90392951	A	C	MDN1	NM_014611.2	c.12002T>G	p.Leu4001Trp
7	1515607	C	G	INTS1	NM_001080453.2	c.5479G>C	p.Ala1827Pro
7	48353932	T	C	ABCA13	NM_152701.4	c.9785T>C	p.Met3262Thr
7	83764200	G	T	SEMA3A	NM_006080.2	c.180C>A	p.Phe60Leu
7	100731045	G	C	TRIM56	NM_030961.2	c.452G>C	p.Gly151Ala
7	103363638	T	A	RELN	NM_173054.2	c.754A>T	p.Ile252Phe
7	105636728	G	A	CDHR3	NM_152750.4	c.641G>A	p.Gly214Glu
7	156742969	G	C	NOM1	NM_138400.1	c.538G>C	p.Glu180Gln
8	2050503	C	T	MYOM2	NM_003970.3	c.2666C>T	p.Ala889Val
8	27729520	G	T	SCARA5	NM_173833.5	c.1419C>A	p.Ser473Arg
8	95952164	C	T	TP53INP1	NM_033285.3	c.397G>A	p.Ala133Thr
8	107719256	C	T	OXR1	NM_001198532.1	c.1510C>T	p.Arg504Cys
9	101983378	G	T	ALG2	NM_033087.3	c.348+451C>A	
9	114860936	C	A	SUSD1	NM_022486.4	c.1288G>T	p.Val430Phe
9	131085558	G	A	COQ4	NM_016035.4	c.202+132G>A	
10	25273728	A	G	ENKUR	NM_145010.3	c.701T>C	p.Met234Thr
10	112262557	G	A	DUSP5	NM_004419.3	c.458G>A	p.Ser153Asn
10	134040354	T	C	STK32C	NM_173575.3	c.589A>G	p.Ile197Val
11	32997064	T	A	QSER1	NM_001076786.2	c.5067+175T>A	
11	66114257	C	T	B4GAT1	NM_006876.2	c.760G>A	p.Ala254Thr
11	108544261	A	G	DDX10	NM_004398.3	c.247+7A>G	
11	110124777	T	C	RDX	NM_001260493.1	c.853A>G	p.Met285Val

12	57963142	G	T	KIF5A	NM_004984.3	c.923G>T	p.Ser308Ile
12	96406993	G	A	LTA4H	NM_000895.2	c.1352C>T	p.Ser451Phe
12	101790158	C	G	ARL1	NM_001177.5	c.515+19G>C	
14	20978732	G	C	RNASE10	NM_001012975.1	c.102G>C	p.Leu34Phe
15	44581522	G	C	CASC4	NM_138423.3	c.295G>C	p.Glu99Gln
16	4457525	G	A	CORO7-PAM16	NM_001201479.1	c.464C>T	p.Ala155Val
16	68379576	A	G	PRMT7	NM_019023.3	c.928-2A>G	
16	84228695	C	T	ADAD2	NM_001145400.1	c.628C>T	p.Gln210Ter
17	27013730	C	G	SUPT6H	NM_003170.4	c.2623C>G	p.Gln875Glu
17	73143689	A	C	JPT1	NM_001002032.2	c.259T>G	p.Ser87Ala
19	11289243	T	C	KANK2	NM_015493.6	c.1403A>G	p.Glu468Gly
19	58867968	A	C	ZNF497	NM_001207009.1	c.1034T>G	p.Leu345Arg
20	2398051	C	T	TGM6	NM_198994.2	c.1510C>T	p.Pro504Ser
22	21133678	T	G	SERPIND1	NM_000185.3	c.78T>G	p.Asp26Glu
X	53279689	T	C	IQSEC2	NM_001111125.2	c.2069A>G	p.Asn690Ser
X	154528117	G	T	CLIC2	NM_001289.5	c.274C>A	p.Gln92Lys

Proband 3

Chr	Position	Ref	Alt	Gene	Accesion	cDNA change	AAA change
1	162337149	C	G	NOS1AP	NM_014697.2	c.1413C>G	p.Asp471Glu
1	243437858	A	G	SDCCAG8	NM_006642.4	c.320A>G	p.His107Arg
2	32249171	G	A	DPY30	NM_032574.3	c.*82C>T	
2	219537459	A	G	STK36	NM_015690.4	c.-89-5A>G	
3	46245507	C	T	CCR1	NM_001295.2	c.298G>A	p.Val100Ile
4	39506886	G	T	UGDH	NM_003359.3	c.1142C>A	p.Ser381Tyr
5	155508	C	G	PLEKHG4B	NM_052909.4	c.2158C>G	p.Gln720Glu
7	1517384	C	T	INTS1	NM_001080453.2	c.4814+5G>A	
7	6085740	T	C	EIF2AK1	NM_014413.3	c.592A>G	p.Ile198Val
7	11441471	C	G	THSD7A	NM_015204.2	c.4362G>C	p.Glu1454Asp
7	48353945	G	T	ABCA13	NM_152701.4	c.9798G>T	p.Leu3266Phe

8	113657433	C	T	CSMD3	NM_198123.1	c.3215G>A	p.Arg1072Lys
9	37518195	G	A	FBXO10	NM_012166.2	c.2441C>T	p.Thr814Ile
11	20078073	C	G	NAV2	NM_001244963.1	c.4901-3C>G	
11	32438969	AC	A	WT1	NM_024426.5	c.965+154delG	
11	119144648	G	A	CBL	NM_005188.3	c.661G>A	p.Ala221Thr
12	121603236	A	G	P2RX7	NM_002562.5	c.610A>G	p.Thr204Ala
13	99378459	T	C	SLC15A1	NM_005073.3	c.163A>G	p.Ile55Val
15	33446640	G	A	FMN1	NM_001277313.1	c.476C>T	p.Pro159Leu
16	2349398	C	T	ABCA3	NM_001089.2	c.1741+6G>A	
18	10752771	C	G	PIEZO2	NM_022068.3	c.3955G>C	p.Gly1319Arg
19	1438627	A	G	RPS15	NM_001018.4	c.4-179A>G	

Proband 4

Chr	Position	Ref	Alt	Gene	Accesion	cDNA change	AAA change
1	34066602	G	T	CSMD2	NM_052896.4	c.6725C>A	p.Pro2242Gln
1	235826323	C	T	LYST	NM_000081.3	c.11323G>A	p.Val3775Met
2	29296732	A	T	PCARE	NM_001029883.2	c.396T>A	p.Asp132Glu
2	58388744	A	AT	FANCL	NM_001114636.1	c.947dupA	p.Tyr316Ter
3	58319318	T	G	PXK	NM_017771.4	c.102+501T>G	
3	113775377	T	A	KIAA1407	NM_020817.1	c.-64A>T	
5	134190813	A	G	C5orf24	NM_001135586.1	c.223A>G	p.Lys75Glu
5	172447309	G	A	ATP6V0E1	NM_003945.3	c.221G>A	p.Trp74Ter
6	33248302	G	A	WDR46	NM_005452.5	c.1430-3C>T	
6	90403863	A	G	MDN1	NM_014611.2	c.9811T>C	p.Ser3271Pro
7	8198233	A	G	ICA1	NM_022307.2	c.629T>C	p.Met210Thr
7	101960927	A	T	SH2B2	ENST00000536178.1	c.1642A>T	p.Ile548Phe
8	99140731	G	A	POP1	NM_001145861.1	c.449G>A	p.Arg150His
8	135649952	A	G	ZFAT	NM_020863.3	c.200T>C	p.Phe67Ser
8	141294090	C	G	TRAPPC9	NM_031466.7	c.2306G>C	p.Ser769Thr
8	145580580	G	A	FBXL6	NM_012162.3	c.772-5C>T	

9	123912675	G	A	CNTRL	NM_007018.4	c.3877G>A	p.Val1293Met
10	126186634	TG	T	LHPP	NM_022126.3	c.564delG	p.Lys189SerfsTer14
11	3690528	G	A	NA10	NM_020402.3	c.260C>T	p.Thr87Ile
12	46320573	C	G	SCAF11	NM_004719.2	c.2911G>C	p.Ala971Pro
12	53186539	C	T	KRT3	NM_057088.2	c.979G>A	p.Ala327Thr
14	23885487	C	G	MYH7	NM_000257.3	c.4679G>C	p.Arg1560Pro
14	104026993	T	A	BAG5	NM_001015049.2	c.632A>T	p.Gln211Leu
15	43816447	G	C	MAP1A	NM_002373.5	c.2776G>C	p.Glu926Gln
17	46114238	G	T	COPZ2	NM_016429.3	c.162C>A	p.Asp54Glu
17	74078723	C	A	ZACN	NM_180990.3	c.1228C>A	p.Pro410Thr
18	11852076	C	T	CHMP1B	NM_020412.4	c.566C>T	p.Ser189Phe
19	1235324	G	A	CBARP	NM_152769.2	c.310+176C>T	
19	6392325	G	A	GTF2F1	NM_002096.2	c.60-340C>T	
19	10602359	C	A	KEAP1	NM_203500.1	c.1219G>T	p.Ala407Ser
19	11352168	C	A	DOCK6	NM_018687.6	c.507C>A	p.His169Gln
19	56813432	T	C	EDDM13	NM_001322061.1	c.-32+11220A>G	
20	4883179	C	T	SLC23A2	NM_203327.1	c.233G>A	p.Ser78Asn
20	61986941	C	A	NA4	NM_000744.6	c.383+386G>T	
20	62896611	G	A	PCMTD2	NM_018257.2	c.411G>A	p.Lys137=
21	16338964	G	A	NRIP1	NM_003489.3	c.1550C>T	p.Thr517Ile
22	38121487	G	C	TRIOBP	NM_001039141.2	c.2924G>C	p.Arg975Pro

Proband 5

**Chr**	**Position**	**Ref**	**Alt**	**Gene**	**Accesion**	**cDNA change**	**AAA change**
1	27099831	G	A	ARID1A	NM_006015.5	c.3716-6G>A	
1	46657778	A	G	POMGNT1	NM_001243766.1	c.1531T>C	p.Tyr511His
2	11362224	C	T	ROCK2	NM_004850.4	c.1084G>A	p.Asp362Asn
2	37480372	G	A	PRKD3	NM_005813.4	c.2621C>T	p.Pro874Leu
2	239237420	G	A	TRAF3IP1	NM_015650.3	c.448G>A	p.Asp150Asn
3	46003741	T	C	FYCO1	NM_024513.3	c.3413A>G	p.Glu1138Gly

3	100988393	G	A	IMPG2	NM_016247.3	c.853C>T	p.Pro285Ser
4	25673262	A	C	SLC34A2	NM_006424.2	c.967A>C	p.Thr323Pro
4	79186240	C	T	FRAS1	NM_025074.6	c.665C>T	p.Ser222Phe
6	31599278	G	A	PRRC2A	NM_004638.3	c.2828G>A	p.Gly943Glu
7	21628848	G	A	DNAH11	NM_001277115.1	c.1996G>A	p.Ala666Thr
9	37541495	C	T	FBXO10	NM_012166.2	c.271G>A	p.Asp91Asn
10	61830422	T	C	ANK3	NM_020987.4	c.10217A>G	p.Asp3406Gly
11	6292649	G	T	CCKBR	NM_176875.3	c.1220G>T	p.Arg407Leu
11	67803807	G	A	NDUFS8	NM_002496.3	c.460G>A	p.Gly154Ser
11	111896976	G	C	DLAT	NM_001931.4	c.334G>C	p.Glu112Gln
11	128786413	C	G	KCNJ5	NM_000890.4	c.1047C>G	p.Asn349Lys
12	65857005	C	T	MSRB3	NM_001193461.1	c.461C>T	p.Ser154Leu
14	92472483	T	C	TRIP11	NM_004239.4	c.1837A>G	p.Ile613Val
16	46729942	T	C	ORC6	NM_014321.3	c.576T>C	p.Asp192=
17	60108953	C	A	MED13	NM_005121.2	c.861G>T	p.Gln287His
18	39618821	G	C	PIK3C3	NM_002647.3	c.2038+7G>C	
19	35829221	A	G	CD22	NM_001771.3	c.1136A>G	p.Glu379Gly
22	36691653	T	A	MYH9	NM_002473.5	c.3383A>T	p.Asn1128Ile

Proband 6

Chr	Position	Ref	Alt	Gene	Accesion	cDNA change	AAA change
1	24124660	T	G	GALE	NM_001127621.1	c.298A>C	p.Lys100Gln
2	71838670	T	C	DYSF	NM_001130987.1	c.4135T>C	p.Cys1379Arg
2	215851347	A	G	ABCA12	NM_173076.2	c.4082T>C	p.Val1361Ala
3	49162043	A	G	LAMB2	NM_002292.3	c.3112T>C	p.Cys1038Arg
3	58121794	G	A	FLNB	NM_001164317.1	c.4853G>A	p.Arg1618His
5	140011796	T	C	CD14	NM_000591.3	c.773A>G	p.His258Arg
6	43013047	G	A	CUL7	NM_001168370.1	c.3208C>T	p.Arg1070Cys
7	2054220	C	T	MAD1L1	NM_001013837.1	c.1276G>A	p.Glu426Lys
14	79434660	G	A	NRXN3	NM_004796.5	c.1994G>A	p.Arg665Gln

14	102471451	G	A	DYNC1H1	NM_001376.4	c.5311G>A	p.Gly1771Arg
15	43023447	G	A	CDAN1	NM_138477.4	c.1822C>T	p.Pro608Ser
16	11643450	G	A	LITAF	NM_001136472.1	c.*43C>T	
17	4718764	C	T	PLD2	NM_002663.4	c.1174-7C>T	
19	11557116	C	T	PRKCSH	NM_002743.3	c.713C>T	p.Pro238Leu
19	19339050	C	T	NCAN	NM_004386.2	c.2621C>T	p.Thr874Met
19	45996636	C	T	RTN2	NM_005619.4	c.815G>A	p.Gly272Glu
19	46376253	A	C	FOXA3	NM_004497.2	c.990A>C	p.Glu330Asp
19	52272353	G	A	FPR2	NM_001005738.1	c.442G>A	p.Gly148Arg
20	57430039	C	T	GNAS	NM_016592.3	c.*42+14098C>T	
22	38524271	C	A	PLA2G6	NM_003560.3	c.1348+5G>T	
